# Exercise and Arterial Stiffness in the Elderly: A Combined Cross-Sectional and Randomized Controlled Trial (EXAMIN AGE)

**DOI:** 10.3389/fphys.2019.01119

**Published:** 2019-09-04

**Authors:** Arne Deiseroth, Lukas Streese, Sabrina Köchli, Romy Sandra Wüst, Denis Infanger, Arno Schmidt-Trucksäss, Henner Hanssen

**Affiliations:** Department of Sport, Exercise and Health, Medical Faculty, University of Basel, Basel, Switzerland

**Keywords:** aging, cardiovascular risk, physical activity, high-intensity interval training, pulse wave velocity

## Abstract

**Introduction:**

Arterial stiffness (AST) is a main determinant of cardiovascular (CV) mortality. Long-term physical activity (PA) is considered to decrease age-related progression of AST but effects of short-term exercise interventions on AST remain unclear.

**Methods:**

In a combined cross-sectional and interventional study approach, we investigated the effects of long-term PA and short-term high-intensity interval training (HIIT) on AST in an older population. 147 older individuals (mean age 59 ± 7 years) were assigned to three groups according to their PA and CV risk profile and compared: healthy active (HA, *n* = 35), healthy sedentary (HS, *n* = 33) and sedentary at risk (SR, *n* = 79). In addition, SR were randomized to either 12 weeks of HIIT or standard recommendations. Pulse wave velocity (PWV) was measured by applanation tonometry. Cardiorespiratory fitness (CRF) was performed by symptom-limited spiroergometry to determine maximal oxygen uptake (VO2max).

**Results:**

Higher CRF was associated with lower PWV (*p* < 0.001) and VO2max explained 18% of PWV variance. PWV was higher in SR (8.2 ± 1.4 m/s) compared to HS (7.5 ± 1.6 m/s) and HA (7.0 ± 1.1 m/s; *p* < 0.001). 12 weeks of HIIT did not change PWV in SR. HIIT-induced reduction in systolic BP was associated with a reduction in PWV (*p* < 0.05).

**Discussion:**

SR show higher PWV compared to HS and long-term PA is associated with lower PWV. Reduction of AST following short-term HIIT seems to depend on a concomitant decrease in blood pressure. Our study puts into perspective the effects of long- and short-term exercise on arterial wall integrity as treatment options for CV prevention in an older population.

**Clinical Trial Registration:**

ClinicalTrials.gov: NCT02796976 (https://clinicaltrials.gov/ct2/show/NCT02796976).

## Introduction

Cardiovascular diseases are responsible for the majority of deaths in western countries and age has been identified as a main risk factor (Piepoli et al., 2016; World Health Organization [WHO], 2018). Vascular tissue biomarkers such as AST provide a means of optimized risk assessment to detect individual subclinical organ damage. Commonly measured as central PWV, AST has gained clinical importance and has been proven to be a reliable predictor for CV risk in the general population (Vlachopoulos et al., 2010). Its addition to standard care can significantly improve CV risk prediction for the individual with a reclassification rate between 13-15% (Vlachopoulos et al., 2010; Ben-Shlomo et al., 2014). Altered PWV indicates subclinical target organ damage and may be used to quantify cumulative damaging effects of CV risk factors on the aging arterial wall integrity.

High CRF is associated with reduced all-cause and CV mortality (Kodama et al., 2009). Previous studies on the effect of regular PA and exercise on indices of AST in the elderly have reported conflicting results (Shibata and Levine, 2012; van Dijk et al., 2015; Ahmadi-Abhari et al., 2017). HIIT is an exercise modality that has attracted attention for its potency to increase CRF and reduce CV risk in patients, for example, with metabolic syndrom (Tjonna et al., 2008).

Data on HIIT and its effects on PWV are scarce. Previous evidence suggests that HIIT may be superior regarding reductions in AST compared to moderate aerobic training in young patients with increased CV risk (Ciolac et al., 2010; Guimaraes et al., 2010). However, a recent meta-analysis by Way et al. (2019) could not detect differences in AST reduction between the two training regimens. The effects of HIIT on AST in elderly people with increased CV risk have not been investigated to date. Our aim was to investigate the associations between long-term PA and central PWV in healthy and diseased elderly. Moreover, we aimed to examine the effects of 12 weeks of HIIT, defined as short-term exercise, on PWV in diseased elderly with clinical indications for add-on exercise treatment.

## Materials and Methods

### Study Design and Subjects

The EXAMIN Age study is a combined cross-sectional and interventional study (Streese et al., 2018). In the cross-sectional part of the study, elderly participants aged between 50 and 80 years were recruited and assigned to three groups according to their PA and CV risk profile: HA, HS, and SR. In the interventional part, SR were randomized into either a walking-based HIIT, performing a supervised training for 12 weeks, or control condition receiving standard PA recommendations only (Piepoli et al., 2016). Participants were recruited by advertisements in local newspapers as well as flyer distribution. Simple randomization was performed by drawing pieces of paper from an envelope by the study physician.

All study visits took place between January 2016 and December 2017. The initial medical screening included a clinical assessment, 24-h blood pressure (BP) measurement and blood sampling. If participants met inclusion criteria, two additional appointments were arranged to perform vascular measurements and assessment of CRF. Follow-up measurements in SR included three identical appointments after 12 weeks. Each participant provided written informed consent and the study design was approved by the local ethics committee and registered in advance. The study has been reported according to the CONSORT standards (Schulz et al., 2010) and was performed according to the Helsinki Declaration for Good Clinical Practice (World Medical Association [WMA], 2013). A detailed study protocol has previously been published (Streese et al., 2018).

### Inclusion and Exclusion Criteria

Inclusion criteria were as follows: Participants were aged between 50–80 years. HA and HS individuals had to be healthy without CV risk factors, whilst SR allocation required at least two of the following CV risk factors: high blood pressure BP (≥140 mmHg systolic or ≥90 mmHg diastolic during 24 h monitoring or antihypertensive medications), obesity (body mass index ≥ 30 kg/m^2^), high fasting plasma glucose levels (≥5.6 mmol/l or antidiabetic medications), high triglyceride levels (≥1.7 mmol/l), low high-density lipoprotein levels (<1.0 mmol/l (male); <1.2 mmol/l (female)), high low-density lipoprotein levels (>4.9 mmol/l or cholesterol-lowering drugs) and current smoker. Additional exclusion criteria for healthy participants were history of CV, pulmonary or chronic inflammatory diseases. Exclusion criteria for individuals at risk were decompensated cardiopulmonary disease and chronic inflammatory diseases as well as compromising orthopedic problems. PA of each participant was judged combining the participant’s PA history, questionnaire-based self-reported PA, objective accelerometers and VO_2__max_. Two sport scientists independently estimated the individual PA level and, if consensus was achieved, participants were assigned to the appropriate group.

### Physical Activity and Cardiorespiratory Fitness

PA was obtained combining self-reported and objective techniques. During the medical examination, participants gave information on past and current PA habits as well as regular sports participation within the last 10 years. A short form of the Freiburg Questionnaire of PA served to calculate metabolic equivalents (METs) based on the Ainsworth Compendium (Frey et al., 1999; Ainsworth et al., 2011). This validated questionnaire allows for estimation of total METs per week as well as METs achieved during sport activities. Objective measurement of daily PA was assured by wearing an Aipermotion 440 accelerometer (Aipermon GmbH, Germany) for six consecutive days. Steps and minutes of walking per day were calculated using the AiperView 440 and ActiCoach MPAT2Viewer Software (Aipermon GmbH, Germany).

Maximal aerobic capacity was obtained by individual ramp protocols on a treadmill ergometry as recommended previously (Myers and Bellin, 2000). The protocol was chosen according to the estimation of the participant’s exercise capacity and were set to reach a test duration of 8–12 min as suggested by the American College of Sport Medicine (Pescatello and American College of Sports Medicine, 2014). VO2max and HR_max_ were recorded for each individual. Gas exchange was assessed using a calibrated breath-by-breath spirometric system (Metalyzer^®^ 3B, MetaSoft^®^, CORTEX Biophysik GmbH, Germany).

### Anthropometry and Blood Sampling

Anthropometric measurements and blood sampling were conducted in the morning under fasting conditions. Body height and body weight were measured to calculate body mass index (kg/m^2^). Body composition was assessed using a standard bio-impedance analyser (InBody 720, Inbody Co., Ltd., South Korea). Blood was drawn by venepuncture of the brachial vein in lithium heparin tubes. Platelet-free plasma was separated by centrifugation (3000 g at room temperature for 10 min), pipetted in aliquots and stored at −80°C for subsequent measurement. High-sensitivity C-reactive protein (hsCRP) was assayed by turbidimetry. 24-h ambulatory BP was obtained by the use of an oscillometric cuff-based sphygmomanometer on the right arm (Mobil-O-Graph^®^, I.E.M GmbH, Germany). Recordings were performed every 20 min during daytime and every 30 min during nighttime.

### Pulse Wave Velocity

Pulse wave velocity was measured according to recommendations of current guidelines (Townsend et al., 2015). To assure standardization, vascular measurements were performed in the morning and participants were asked to refrain from exercise 24 h and from alcohol and caffeine consumption 12 h prior to the examination. Participants had to rest 10 min in a supine position after systolic and diastolic BP at rest were taken with a cuff from the right brachial artery, using an automatic BP monitor system (Omron Healthcare, Germany). PWV was measured using a standard device by use of applanation tonometry (SphygmoCor CPV^®^, ATCor Medical, Australia). High quality measurements with a deviation in pulse waveforms of less than 10% within 10-second recordings were considered valid. The mean value of two valid measurements with a mean difference of ≤1 m/s was used for further calculations. Central PWV in m/s was calculated as distance divided by transit time of carotid and femoral pulse wave (foot-to-foot method). The distance for carotid-femoral PWV was determined by subtracting the suprasternal notch (SSN) to the carotid site distance from the SSN to the femoral site. All analyses were performed by the same experienced investigator who was blinded for group allocation.

### Exercise Intervention

In the interventional part of the study, a 12 week nordic walking-based HIIT, was applied in the SR group three times per week. Training sessions were supervised by sport scientists. Stepwise increase of intensity in the first two weeks was conducted to familiarize former sedentary participants with aerobic exercise. After two weeks, 4 × 4 min of HIIT at an intensity of 80–90% of HR_max_ was performed. Recovery time between each interval lasted 3 min and was set at an intensity of 60–70% HR_max_. Including warm-up and cool-down, an average training session lasted 60 min.

### Statistical Analysis and Sample Size Calculation

Participants’ characteristics are presented with mean [standard deviation (SD)] for continuous variables and frequency counts for categorical variables. Analysis of variance (ANOVA) was used to detect overall group differences in the cross-sectional approach. Multiple linear regression was performed to identify between group differences adjusting for possible confounders. The assumptions of the regressions were verified using residual plots. In the interventional approach, we performed an analysis of covariance (ANCOVA) to detect and quantify differences between HIIT and control group adjusted for baseline values (Vickers and Altman, 2001). A multiple linear regression model was used to describe factors explaining the difference in central PWV following HIIT. We used two-sided tests with a significance level of 5% in all our analyses. Data were analyzed using R (Version 3.5.1^1^).

Details on sample size calculation have previously been published in the study protocol and were performed for the cross-sectional and interventional approach separately (Streese et al., 2018). For the cross-sectional part we assumed that the expected central PWV corresponds to 8.5, 9.5, and 11.5 m/s for HA, HS, and SR, respectively, and that the standard deviation given any particular group is 1.5 m/s (Tedesco et al., 2004; Gando et al., 2010). Central PWV was the main outcome and an 80% power on a 2-sided significance level of 0.05 was targeted. This resulted in 36 participants needed for each group in the cross-sectional approach. For the interventional part of the study, we assumed that the expected difference in central PWV after 12 weeks between SR in the intervention and those in the control group is 1.0 m/s and that the standard deviation is 1.5 m/s (Madden et al., 2009). This led to the calculation of a minimum number of 36 participants. Taking dropouts into account we aimed to reach 40 subjects in the HA and in the HS group and 80 persons in the SR group (40 intervention and 40 controls). For sample size calculation, we used the POWER and GLMPOWER procedure in SAS 9.3 (SAS Institute Inc., Cary, NC, United States).

## Results

The data underlying the study are available from the corresponding author upon reasonable request.

### Participants

**FIGURE 1:**
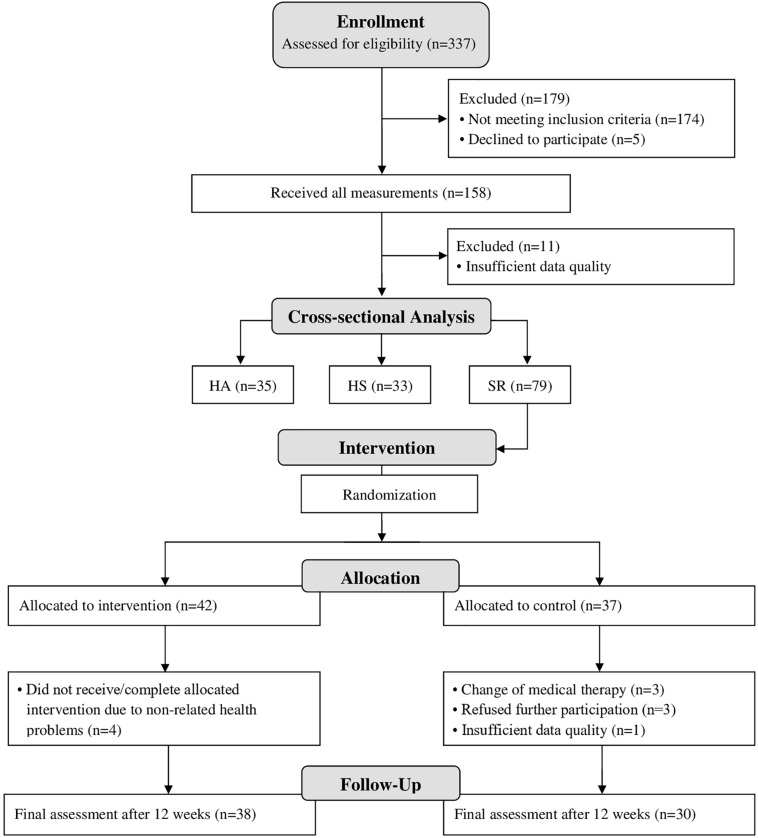
Flow-chart.

The recruitment process is summarized in a CONSORT flow diagram (Figure 1). In the cross-sectional part, 35 persons were included in the HA group, 33 in the HS group and 79 in the SR group. Eighty-six percent of the SR group were obese, 70% were hypertensive, 56% suffered from dyslipidaemia, 41% were diabetics and 34% were smokers (Table 1). The participants’ characteristics of all three groups including CV risk factors, PA and CRF data as well as the vascular indices are presented in Table 2.

**TABLE 1:** Risk factors in sedentary at risk.

	***n***	**%**
**Obesity**	68	86
**High Blood Pressure**	55	70
SBP ≥ 140 mmHg or DBP ≥ 90 mmHg (24 h)	25	32
Antihypertensive medication	36	46
**Dyslipidaemia**	44	56
LDL > 4.9 mmol/l	2	3
HDL < 1.0 mmol/l (male) or <1.2 mmol/l (female)	18	23
Triglycerides > 1.7 mmol/l	29	37
Cholesterol lowering medication	13	16
**Diabetes**	32	41
Fasting glucose ≥ 5.6 mmol/l	32	41
Antidiabetic medication	7	9
**Smoking**	27	34

*SBP, systolic blood pressure; DBP, diastolic blood pressure; LDL, low-density lipoprotein, HDL, high-density lipoprotein.*

**TABLE 2:** Participants’ characteristics.

	**HA (*n* = 35)**	**HS (*n* = 33)**	**SR (*n* = 79)**	***p***
**Clinical data**				
Age, yr	60 ± 7	59 ± 7	58 ± 6	0.325
Female sex, n (%)	17 (49)	24 (73)	41 (52)	0.080^∗^
Height, cm	171 ± 7.7	168 ± 8.8	169 ± 8.0	0.403
Body mass, kg	64.4 ± 6.6	70.7 ± 10.2	95.5 ± 13.9	<0.001
BMI, kg/m^2^	22.2 ± 1.7	24.9 ± 2.5	33.4 ± 4.0	<0.001
WC, cm	82 ± 7	89 ± 9	112 ± 12	<0.001
HR, bpm	62 ± 8	77 ± 12	79 ± 11	<0.001
SBP at rest, mmHg	128 ± 16	128 ± 15	132 ± 15	0.317
DBP at rest, mmHg	78 ± 8	81 ± 8	88 ± 10	<0.001
PP at rest, mmHg	50 ± 12	46 ± 11	44 ± 12	0.052
24H SBP, mmHg	119 ± 6	121 ± 7	130 ± 11	<0.001
24H DBP, mmHg	76 ± 6	76 ± 6	81 ± 8	<0.001
Fasting glucose,	4.6 ± 0.4	4.7 ± 0.5	5.7 ± 1.8	<0.001
mmol/l				
Triglyceride, mmol/l	0.92 ± 0.28	1.09 ± 0.31	1.80 ± 1.11	<0.001
HDL, mmol/l	1.99 ± 0.41	1.69 ± 0.38	1.30 ± 0.32	<0.001
LDL, mmol/l	2.85 ± 0.75	3.2 ± 0.83	3.23 ± 0.79	0.064
hsCRP, mg/l	0.9 ± 1.0	1.7 ± 2.3	3.7 ± 4.2	<0.001
**Physical activity and fitness**				
Total PA, MET/week	171 ± 8.4	135 ± 56	126 ± 55	<0.001
Sport activity,	47 ± 37	2 ± 2	1 ± 3	<0.001
MET/week				
Steps per day, n	13800 ± 4629	10222 ± 3213	9028 ± 3283	<0.001
Walking per day, min	31 ± 25	19 ± 13	14 ± 11	<0.001
VO_2__max_, ml/min/kg	42.6 ± 8.2	29.7 ± 4.0	26.1 ± 4.4	<0.001
**Arterial Stiffness**				
Central PWV, m/s	7.0 ± 1.1	7.5 ± 1.6	8.2 ± 1.4	<0.001

*Data are mean ± standard deviation. ANOVA was used to calculate overall group differences. ^∗^Chi-squared test was used. HA, healthy active; HS, healthy sedentary; SR, sedentary at risk; BMI, body mass index; WC, waist circumference; HF, heart rate; SBP, systolic blood pressure; DBP, diastolic blood pressure; HDL, high-density-lipoprotein, LDL, low-density-lipoprotein; hsCRP, high-sensitivity C-reactive Protein; PA, physical activity; MET, metabolic equivalent; VO_2max_, maximal oxygen uptake; PWV pulse wave velocity.*

### Cross-Sectional Part

**FIGURE 2:**
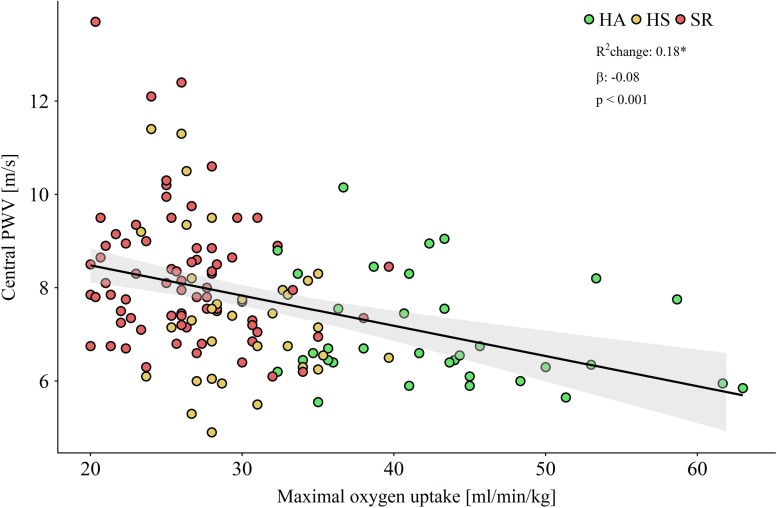
Scatterplot showing maximal oxygen uptake by central pulse wave velocity (PWV) for healthy active (HA) and sedentary (HS) as well as sedentary at risk (SR). Regression line and 95% confidence intervall of mean standard deviation are visualized. Multiple linear regression was adjusted for age and sex.

**FIGURE 3:**
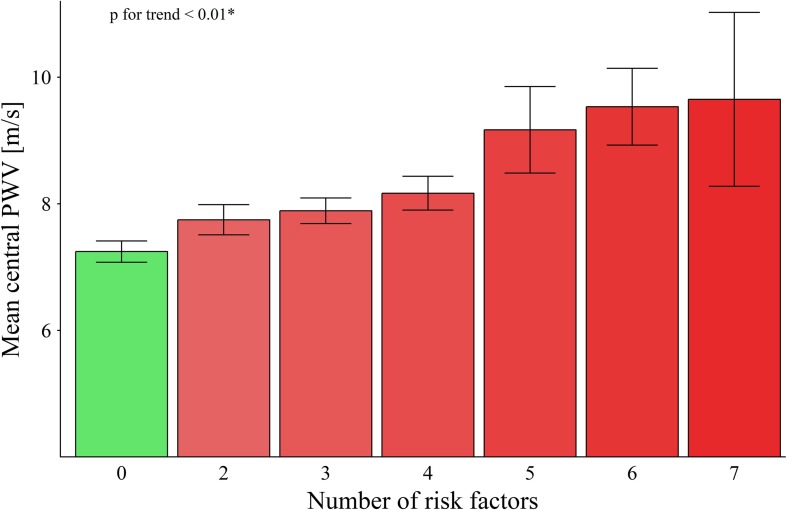
Number of risk factors in participants and the corresponding mean central pulse wave velocity (PWV). ^∗^Jonckheere Trend Test.

**FIGURE 4:**
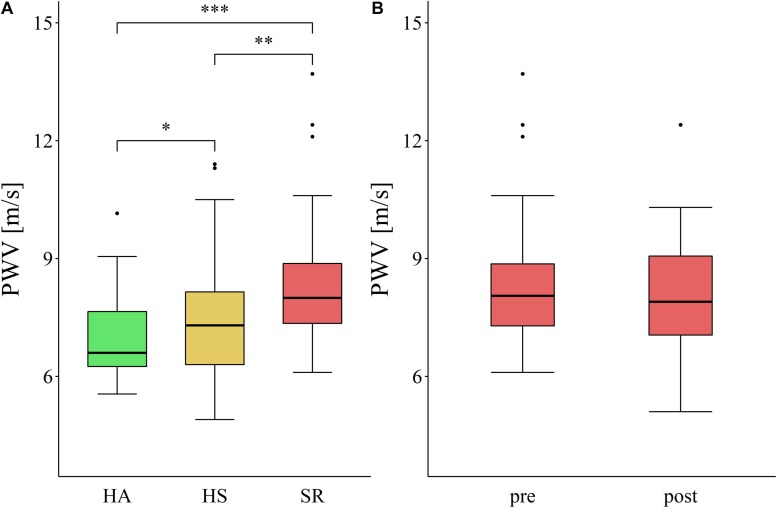
Central pulse wave velocity in the cross-sectional **(A)** and interventional **(B)** approach. HA, healthy active; HS, healthy sedentary; SR, sedentary at risk; ^∗^*p* < 0.05; ^∗∗^*p* < 0.01; ^∗∗∗^*p* < 0.001.

Overall higher CRF was significantly associated with lower central PWV (*p* < 0.001) and VO_2__max_ explained 18% of the variance in PWV in all participants after adjustment for age and sex (Figure 2). An increase of 10 ml/min/kg in VO_2__max_ was associated with a decrease of PWV by 0.8 m/s (*p* < 0.001). PWV increased with an increasing number of risk factors (*p* for Trend < 0.01; Figure 3). In the between group comparison, PWV was highest in SR (8.2 ± 1.4 m/s) compared to HS (8.2 ± 1.4 m/s) and HA (7.0 ± 1.1 m/s; Table 3). ANOVA revealed a significant overall group difference (*p* < 0.001). Multiple linear regression revealed significant differences in PWV between all groups (Figure 4A and Table 4).

**TABLE 3:** Population characteristics in sedentary at risk before and after HIIT.

**Outcome**	**Adjusted**	**Lower**	**Upper**	***p***
	**difference^∗^**	**CI (95%)**	**CI (95%)**	
**Anthropometric data**				
Weight, kg	–0.4	–1.5	0.7	0.45
BMI, kg/m2	–0.2	–0.6	0.2	0.38
WC, cm	–1.2	–4.2	1.7	0.41
Fat mass,%	–2.0	–3.6	–0.5	0.01
Muscle mass,%	1.0	0.2	1.9	0.02
HR, bpm	–2.9	–7.6	1.7	0.21
SBP at rest, mmHg	–1.0	–6.0	4.7	0.80
DBP at rest, mmHg	1.6	–2.2	5.5	0.39
**Cardiorespiratory fitness**				
VO_2__max_, ml/min/kg	3.4	2.5	4.3	<0.001
**Arterial Stiffness**				
Central PWV, m/s	0.1	–0.3	0.6	0.60
Central PWV^†^, m/s	0.2	–0.2	0.6	0.29

*^∗^Analysis of covariance was used to detect differences of post- versus pre-intervention values between intervention and control condition. ^†^Additional adjustment for delta of systolic blood pressure (pre- minus post-value). CI, confidence interval; BMI, body mass index; WC, waist circumference; HR, heart rate; SBP, systolic blood pressure; DBP, diastolic blood pressure; PWV, pulse wave velocity.*

**TABLE 4:** Between group differences in central pulse wave velocity.

	**Model**	**adj. R^2^**	**Mean difference (95% CI)**	***p***
HA to HS	1	0.34	0.64 (0.07, 1.20)	0.027
	2	0.38	0.63 (0.08, 1.18)	0.026
HS to SR	1	0.26	0.79 (0.25, 1.32)	0.004
	2	0.28	0.70 (0.17, 1.23)	0.010
HA to SR	1	0.32	1.37 (0.90, 1.84)	<0.001
	2	0.33	1.33 (0.86; 1.81)	<0.001

*Multiple linear regression for group differences central pulse wave velocity (m/s). Model 1: adjusted for age and sex. Model 2: adjusted for age, sex and systolic blood pressure. Abbreviations: adj., adjusted; CI, confidence interval; HA, healthy active; HS, healthy sedentary; SR, sedentary at risk.*

### Interventional Part

The final analysis was performed in 38 persons of the intervention group and 30 persons of the control group (Figure 1). After 12 weeks of HIIT, CRF improved significantly, but this was not accompanied by significant changes in central PWV (Table 3 and Figure 4B).

**FIGURE 5:**
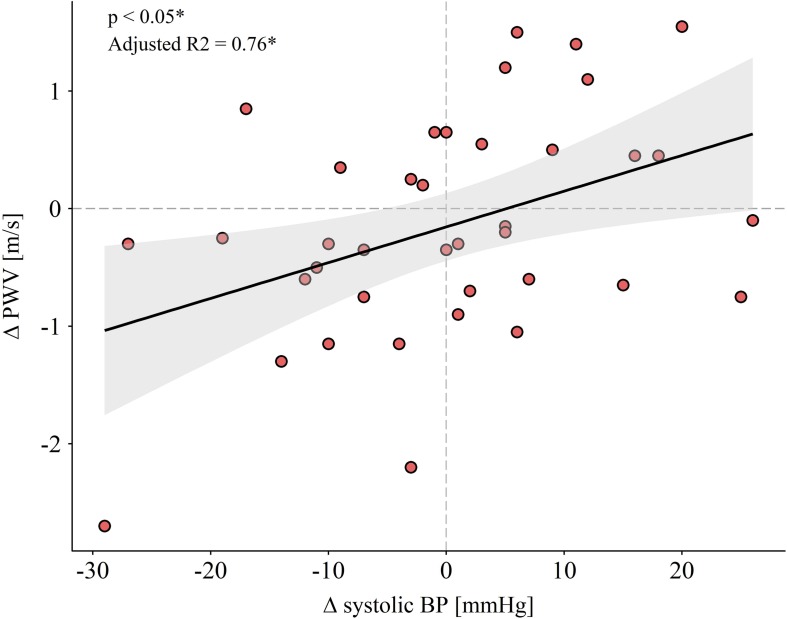
Regression line and 95% confidence interval of standard deviation are visualized. Deltas were calculated subtracting pre- from post-value. ^∗^Multiple linear regression adjusted for age and sex, baseline central PWV and body mass index. Abbreviations: BP, blood pressure; PWV, pulse wave velocity.

Adjustment for differences in systolic BP (pre- to post-training) did not change these results. Pre- and post-values for all measurements in the intervention and the control group are presented in Table 5. Main determinants for the adaptations of PWV after HIIT were the baseline PWV (*p* < 0.001) and changes in systolic BP (*p* < 0.018). The association of changes in systolic BP with changes in central PWV is shown in Figure 5.

**TABLE 5:** Population characteristics in sedentary at risk before and after HIIT.

	**Intervention (*n* = 38)**		**Control (*n* = 30)**	
**Outcome**	**Pre**	**Post**	**Pre**	**Post**
**Clinical data**				
Age, year	58 ± 5		57 ± 6	
Female sex, *n* (%)	18		19	
Weight, kg	95.1 ± 12.3	93.9 ± 12.6	94.4 ± 14.8	93.6 ± 14.5
BMI, kg/m^2^	33.3 ± 3.0	32.8 ± 3.2	33.1 ± 5.1	32.8 ± 5.0
WC, cm	111 ± 9	109 ± 10	110 ± 14	110 ± 14
Fat mass,%	40 ± 8	38 ± 8	42 ± 8	42 ± 7
Muscle mass,%	32 ± 7	33 ± 7	30 ± 6	30 ± 5
HR, bpm	79 ± 12	74 ± 11	76 ± 9	75 ± 11
SBP at rest, mmHg	133 ± 14	134 ± 12	128 ± 14	131 ± 15
DBP at rest, mmHg	88 ± 10	87 ± 7	86 ± 10	85 ± 11
24h SBP, mmHg	130 ± 10	132 ± 12	128 ± 10	126 ± 11
24h DBP, mmHg	82 ± 7	83 ± 8	79 ± 8	79 ± 9
Fasting glucose, mmol/l	5.8 ± 2.2	5.7 ± 1.8	5.5 ± 1.1	5.5 ± 1.1
Triglyceride, mmol/l	1.82 ± 1.03	1.87 ± 1.13	1.62 ± 0.79	1.82 ± 1.00
HDL, mmol/l	1.29 ± 0.3	1.29 ± 0.28	1.37 ± 0.31	1.35 ± 0.34
LDL, mmol/l	3.34 ± 0.83	3.08 ± 0.90	3.03 ± 0.69	2.92 ± 0.78
hsCRP, mg/l	3.3 ± 2.5	3.0 ± 2.2	4.1 ± 6.1	4.4 ± 7.8
**Cardiorespiratory fitness**				
VO_2max_, ml/min/kg	26.4 ± 3.9	28.6 ± 1.1	26.2 ± 5.1	25.1 ± 4.2
**Arterial Stiffness**				
Central PWV, m/s	8.2 ± 1.2	8.1 ± 1.1	8.2 ± 1.6	7.9 ± 1.6

*Data are mean ± standard deviation. SD, standard deviation; BMI, body mass index; WC, waist circumference; HR, heart rate; SBP, systolic blood pressure; DBP, diastolic blood pressure; HDL, high-density lipoprotein; LDL, low-density lipoprotein; hsCRP, high-sensitivity C-reactive Protein; PWV, pulse wave velocity.*

## Discussion

Our study results demonstrate the importance of long-term PA and the limited impact of short-term exercise training on large artery stiffness in an older population. Long-term PA was associated with lower central PWV even in the absence of CV risk factors. PWV was higher in SR compared to HS. Overall, higher VO_2__max_ and fewer CV risk factors were associated with lower PWV. Most importantly, 12-weeks of HIIT did not reduce PWV in elderly at increased CV risk.

Aging is characterized by continuous remodeling of the arterial wall and Vaitkevicius et al. (1993) were the first to suggest that higher CRF may mitigate stiffening of the aging arterial tree. In our study, with every 10 ml/min/kg increase in VO_2__max_, PWV dropped by 0.8 m/s. Active participants presented with 0.5 m/s lower central PWV than their sedentary counterparts. An increase of 1 m/s in central PWV has been associated with a 15% risk increase in CV and all-cause mortality (Vlachopoulos et al., 2010). Thus, our cross-sectional findings indicate an 8% risk increase attributable to a sedentary lifestyle even in healthy elderly.

Whether short-term exercise can postpone or even reverse the age- and disease- related progression of AST remained unresolved to date. HIIT has been shown to be effective in reducing CV risk in patients with metabolic syndrome (Tjonna et al., 2008). Only a few studies have evaluated the effects of HIIT on central PWV and these produced conflicting results. While Ciolac et al. (2010) and Guimaraes et al. (2010) were able to show a reduction of AST in response to HIIT in young individuals with increased CV risk, these findings could not be reproduced in healthy older persons by Kim et al. (2017). Our study is the first to examine the effects of 12 weeks of HIIT in sedentary elderly with CV disease, demonstrating that high-intensity exercise training does not improve large artery stiffness in older individuals. Several systematic reviews and meta-analyses with inconsistent findings exist on the effects of exercise interventions on AST, but none of them sets focus on elderly people (Ashor et al., 2014; Montero et al., 2014a, b; Huang et al., 2016). Our results indicate a reduced vascular adaptability in older individuals and highlight the pivotal role of age when assessing the effect of exercise training on AST.

This is underlined by our cross-sectional data showing that sustained long-term habitual PA is associated with better vascular function. This raises the question whether a threshold exercise duration may exist in order to achieve improvement of AST in elderly with CV disease. The two studies reporting reduced PWV after HIIT lasted 16 weeks but were conducted in younger individuals (Ciolac et al., 2010; Guimaraes et al., 2010). In sedentary elderly persons, 1 year of aerobic exercise at moderate intensity did not reduce AST (Shibata and Levine, 2012). Moreover, it has previously been argued that significant improvements in VO_2__max_ are prerequisite for relevant reductions in PWV after short-term exercise (Huang et al., 2016). However, in our study HIIT induced a significant increase of CRF without improving PWV in elderly with CV disease. In conjunction with the previous literature and our current findings it becomes evident that sustained long-term PA may be needed to improve, postpone or even reverse the increased PWV in elderly with CV disease.

Montero et al. (2014b) reported that a reduction in central PWV was dependent on concomitant reductions in systolic BP independent of structural changes in the arterial wall. From a physiological point of view, close associations between BP and PWV are to be expected (Kim et al., 2007). Indeed, our intervention group showed significant associations between changes in central PWV and systolic BP which is in-line with the findings of Montero et al. (2014b). In addition, 46% of our participants in the SR group were already treated for hypertension. In elderly persons pretreated for high BP, additional HIIT may have limited effects on further BP reductions, and the arterial capacity to adapt to exercise stimuli may be diminished.

To put our findings into perspective and add to the debate on the effects of exercise on AST, possible underlying mechanisms need to be discussed. Vascular wall integrity is defined by functional as well as structural properties of the arterial wall (Zieman et al., 2005). Functional changes are largely dependent on vascular smooth muscle tone which is regulated locally by endothelial function. Structural properties depend, in large part, on the collagen and elastin composition of the vascular wall. Recently, the role of exercise as a modulator of AST in the context of the potential mechanisms involved has been addressed (Sacre et al., 2014). Though exercise affects functional as well as structural components of the arterial wall, Sacre et al. (2014) suggested that longer-term exercise would be needed to provoke structural changes in the arterial wall. Our results support this postulation. This seems to be the case for older age in particular, where long lasting structural elastin degradation and collagen deposition cannot be reversed by relative short-term high-intensity exercise interventions. Interestingly, we previously demonstrated that a 12-week high-intensity exercise intervention in the same participants increased arteriolar and decreased venular retinal vessel diameters (Streese et al., 2019), which is associated with reduced CV and all-cause mortality (McGeechan et al., 2009; Seidelmann et al., 2016). These improvements in retinal vessel diameters were associated with reduced oxidative stress, triggered by a restoration of p66^*Shc*^ promoter methylation and subsequent reduced p66^*Shc*^ expression. Based on these results, it can be assumed that the retinal vessel analysis is a more sensitive screening tool to detect short-term high-intensity exercise effects compared to measurements of central PWV.

Few limitations have to be addressed. Our sample size is relatively small. However, the sample size calculation based on PWV as the primary outcome has been reached, except for the control group of patients. Our study shows lack of PWV reductions after 12 weeks of HIIT in elderly subjects at CV risk. Whether interventions of 16 weeks or longer may lead to significant PWV reductions remains unclear. A threshold duration cannot be estimated on the basis of our data. Furthermore, an unbalanced sex distribution is evident in HS. This may have tempered between group differences. However, sex does not account for normal values of PWV according to current recommendations (Reference Values for Arterial Stiffness Collaboration, 2010). Our results were adjusted for sex to minimize potential confounding and improve the proportion of explained variance. In addition, only sedentary persons at increased CV risk performed HIIT, as this group had a clear indication for exercise therapy and was expected to benefit most from aerobic exercise. Our Patients were characterized by a number of treated and untreated CV diseases that may have differential impacts on the arterial wall. We believe this represents the real life setting, as elderly persons are increasingly prone to comorbidities.

## Conclusion

CRF is a main determinant of central PWV in an aging population. Our results demonstrate that long-term active compared to sedentary lifestyle is associated with lower AST even in healthy elderly. This suggests that age- and disease-related vascular stiffening and the associated worse CV outcome can be postponed by long-term regular PA. Short-term exercise, even at higher intensities, cannot improve arterial stiffening in sedentary elderly with increased CV risk. Exercise-induced reductions of AST seem to depend on a concomitant decrease of BP. The results of our study shed light on the influence of long-term PA, CRF and the effects of short-term exercise on arterial stiffening as treatment options for CV disease prevention in an older population.

## Data Availability

The raw data supporting the conclusions of this manuscript will be made available by the authors, without undue reservation, to any qualified researcher.

## Ethics Statement

The studies involving human participants were reviewed and approved by the Ethics Committee of Northwest and Central Switzerland (EKNZ 2015-351). The patients/participants provided their written informed consent to participate in this study.

## Author Contributions

AD drafted the manuscript, and performed the stiffness examinations and all medical assessments. LS was responsible for the general data collection and anthropometric measurements, organized the training, and critically revised the manuscript. SK revised the manuscript and discussed the methodological approach. RW was involved in the data collection and supervised the training. DI gave statistical support and revised the manuscript. AS-T critically revised the manuscript. HH is principal investigator, designed the study, critically discussed the results, and critically revised the manuscript. All authors read and approved the final manuscript.

## Conflict of Interest Statement

The authors declare that the research was conducted in the absence of any commercial or financial relationships that could be construed as a potential conflict of interest.

## Footnotes

1 www.r-project.org

## Abbreviations

AST: arterial stiffness
CRF: cardiorespiratory fitness
CV: cardiovascular
EXAMIN Age, Exercise: Arterial Crosstalk-Modulation and Inflammation in an Aging Population
HA: healthy active
HIIT: high-intensity interval training
HR_max_: maximum heart rate
HS: healthy sedentary
PA: physical activity
PWV: pulse wave velocity
SR: sedentary individuals with increased CV risk
SSN: suprasternal notch
VO2_max_: maximal aerobic capacity.
